# NY-ESO-1 expression in DCIS: A new predictor of good prognosis

**DOI:** 10.18632/oncoscience.348

**Published:** 2017-04-28

**Authors:** R. Charles Coombes, Otavia L. Caballero, Sami Shousha, Sadaf Ghaem-Maghami, Laura Woodley-Barker, Charlotte S. Wilhelm-Benartzi, A. Munro Neville

**Affiliations:** ^1^ Imperial College Healthcare NHS Trust & Imperial College, London, Hammersmith Hospital, London, UK; ^2^ Ludwig Collaborative Laboratory, Ludwig Institute for Cancer Research, Department of Neurosurgery, The Johns Hopkins University School of Medicine, Baltimore, MD, USA; ^3^ Imperial College Healthcare NHS Trust & Imperial College, London, Charing Cross Hospital, London, UK; ^4^ Centre for Trials Research, College of Biomedical and Life Sciences, Cardiff University, Cardiff, UK; ^5^ Ludwig Institute for Cancer Research, New York, NY, USA

**Keywords:** CT antigen, breast cancer, immunotherapy, prognosis, ductal-carcinoma-in-situ (DCIS)

## Abstract

**Background:**

At present, it is difficult to predict which patients with ductal carcinoma-in-situ (DCIS) will subsequently develop frank invasive breast cancer (IDC). A recent survey by our group has shown that NY-ESO-1 and MAGEA are both expressed in DCIS. This study was aimed at determining whether expression of these antigens was related to the later development of IDC.

**Results:**

14 of 42 (33%) of patients developed invasive breast cancer during the follow up period. Only one of those DCIS cases that relapsed was positive for NYESO-1 at diagnosis. In contrast, DCIS samples of 15 of the 28 (54%) of those patients who remained disease-free expressed NY-ESO-1. (Permutation chi square p=0.0033).

**Methods:**

We identified 42 patients with DCIS, and followed them up for more than 10 years. NY-ESO-1 and MAGEA were demonstrated by immunostaining as were CD8+ infiltrates on all sections together with the conventional markers, ER, PR, and HER2.

**Conclusions:**

Expression of NY-ESO-1 may predict those patients who will not subsequently develop invasive breast cancer and could therefore potentially be helpful in defining prognosis in patients with DCIS.

## INTRODUCTION

Mammographic screening of healthy women has resulted in the increased detection of ductal carcinoma *in Situ* (DCIS), such that one-fifth of all screen detected breast cancers are now so diagnosed [1]. Thus, the incidence has more than quadrupled in women of screening age in the Western World. Many studies have attempted to determine prognostic indices that could assist clinicians in their decision-making. To date, nuclear grade and activity, comedo necrosis and HER-2 positivity are the prognostic signs frequently used in helping to decide whether conservative surgery or mastectomy is the preferred surgical treatment, and whether post-operative treatment should be administered [2, 3].

Cancer Testis (CT) genes are not normally expressed in adult tissues but are expressed in the human germ line and are activated in various malignancies. Because of their restricted expression pattern, CT proteins are frequently immunogenic in cancer patients and therefore they are considered important targets for anticancer immunotherapy [4]. Using publicly available gene expression datasets to interrogate the expression of CT genes in breast tumors, we have demonstrated that while a minority of unselected breast cancers expressed CT genes, a significantly higher expression frequency was detected in estrogen (ER) and progesterone receptor (PR) negative breast cancer cell lines and primary breast carcinomas [5]. Immunohistochemical studies confirmed the association of CT antigen expression and ER negativity in breast tumors and demonstrated their frequent expression in tumors with higher nuclear grade [6, 7].

Recently our group has explored the expression of CT antigens in DCIS. We found that many samples of DCIS expressed a variety of CT antigens; amongst those for which antibodies were available for immunohistochemistry, we found that NY-ESO-1 was expressed in a high proportion of DCIS tissues, especially those that were ER negative [8].

This paper describes the extension to this study. Employing an independent set of patients with a documented follow-up period, we have explored the relationship between NY-ESO-1 expression and subsequent development of invasive breast cancer. The data show that lack of NY-ESO-1 expression confers a higher risk of developing invasive breast cancer in this cohort of patients.

## RESULTS

### Univariate characteristics

Patients were followed up for a mean of 10.54 years (Table 1). 12/42 (29%) patients had histologically assessed high-grade DCIS, with the remaining patients having intermediate or low-grade DCIS. The majority of patients (86%) received radiotherapy after surgery. No patient received systemic therapy after excision of DCIS.

**Table 1:** Demographic characteristics

Demographic characteristics (N=42)	N (%) or Mean (SD)
**Follow up time (years)**	
Mean (SD)	10.54 (5.29)
**Recurrence time (years)**	
Mean (SD)	10.56 (5.32)
**Death time (years)**	
Mean (SD)	12.70 (4.29)
**Age**	
Mean (SD)	73.96 (8.0)
**Invasive disease/Recurrence**	
No	28 (67%)
Yes	14 (33%)
**Death**	
No	29 (69%)
Yes	13 (31%)
**DCIS type**	
High grade	12 (29%)
Intermediate grade	11 (26%)
Low grade	16 (38%)
Missing	3 (7%)
**Radiotherapy**	
No	6 (14%)
Yes	36 (86%)

NY-ESO-1 and MAGEA were expressed in 38% and 17% of cases, respectively. Positive staining was where all or a plurality of the tumour cells were stained. CD8+ lymphocytes were detected in 96% of cases and were classified into three grades as previously described [8] (Table 2). We found positive results for ER, PR and HEtR2 in 60%, 50% and 19% of the cases, respectively.

**Table 2:** Marker characteristics

Marker characteristics (N=42)	N (%)
**NY-ESO-1_E978**	
Negative	25 (60%)
Positive	16 (38%)
Missing*	1 (2%)
**MAGEA_6C1**	
Negative	34 (81%)
Positive	7 (17%)
Missing *	1 (2%)
**CD8**	
1+	18 (43%)
2+	9 (21%)
3+	13 (31%)
Negative	1 (2%)
Missing*	1 (2%)
**CD8 recoded**	
Negative or 1+	19 (45%)
2+ or 3+	22 (52%)
Missing*	1 (2%)
**ER positive**	
No	11 (26%)
Yes	25 (60%)
Missing**	6 (14%)
**PgR positive**	
No	15 (36%)
Yes	21 (50%)
Missing**	6 (14%)
**Her2 positive**	
No	25 (60%)
Yes	8 (19%)
Missing**	9 (21%)

*material detached at staining.

**no tumour found on recutting the sections.

### Univariate associations to invasive disease

During the follow-up period, 14 of 42 (33%) patients developed IDC: only 1 case of those DCIS cases that relapsed was positive for NY-ESO-1 at diagnosis (Table 3). In contrast, DCIS samples of 15 of the 28 (54%) of those patients who remain disease-free expressed NY-ESO-1 (permutation chi square p=0.0033). Expression of NY-ESO-1 was significantly associated with 94% lower odds of having invasive disease (Table 3). In contrast to the good predictive effect of NYESO-1, MAGEA expression appeared to correlate with subsequent IDC development. The presence of CD8+ lymphocytes was not predictive of development of invasive disease.

**Table 3:** Table of markers by Recurrence/Invasive disease

Marker characteristics (N=42)	Recurrence/Invasive disease		P-value*	OR**	95% CI**	P-value**	HR***	95% CI***	P-value***	Logrank P-value
	No (n=28)	Yes (n=14)								
**NY-ESO-1_E978**										
Negative (n=25)	12	13	0.0033	0.061	(0.003, 0.376)	0.0118	0.138	(0.018, 1.065)	0.0575	0.0262
Positive (n=16)	15	1								
Missing (n=1)	1	0								
**MAGEA_6C1**										
Negative (n=34)	24	10	0.0772	3.2001	(0.602, 18.89)	0.172	3.2776	(0.998, 10.76)	0.0503	0.0383
Positive (n=7)	3	4								
Missing (n=1)	1	0								
**CD8**										
1+ (n=18)	11	7	0.8056	1.0526	(0.511, 2.162)	0.887	1.171	(0.669, 2.051)	0.579	0.717
2+ (n=9)	7	2								
3+ (n=13)	8	5								
Negative (n=1)	1	0								
Missing (n=1)	1	0								
**CD8 recoded**										
Negative or 1+ (n=19)	12	7	0.5067	0.8	(0.215, 2.950)	0.735	1.053	(0.368, 3.013)	0.923	0.923
2+ or 3+ (n=22)	15	7								
Missing (n=1)	1	0								
**ER positive**										
No (n=11)	8	3	0.6848	1.037	(0.220, 5.766)	0.964	1.071	(0.276, 4.157)	0.922	0.922
Yes (n=25)	18	7								
Missing (n=6)	2	4								
**PgR positive**										
No (n=15)	9	6	0.1465	0.353	(0.073, 1.550)	0.174	0.4605	(0.130, 1.636)	0.231	0.219
Yes (n=21)	17	4								
Missing (n=6)	2	4								
**Her2 positive**										
No (n=25)	19	6	0.0739	3.1667	(0.590, 17.69)	0.174	2.5134	(0.705, 8.966)	0.156	0.142
Yes (n=8)	4	4								
Missing (n=9)	5	4								

*P-values were computed using a permutation chi square test using 10,000 permutations.

**Univariate logistic regression was used to derive these values.

^^As continuous variable.

***Univariate cox proportional hazards regression was used to derive these values.

**Figure 1:**
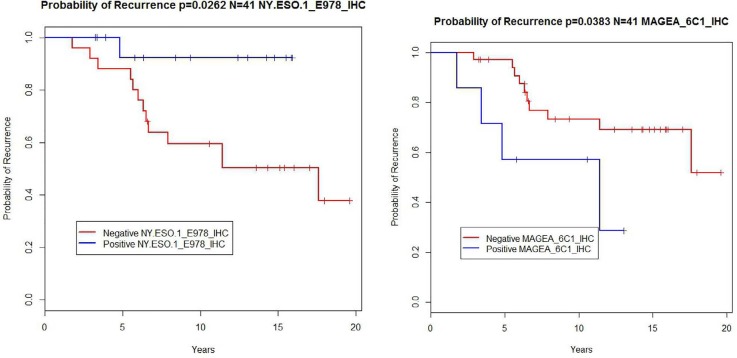
Kaplan Meier plot of probability of recurrence by NY-ESO-1 and by MAGEA status

Kaplan-Meier plots were constructed for all markers under study. Only NY-ESO-1 and MAGEA had a significant likelihood ratio test (LRT) p-value at 0.0262 and 0.0383 respectively to predict better outcome in the case of NY-ESO-1 and worse outcome in the case of MAGEA (Table 3 and Figure 1).

### Association between the markers

There was no relationship between NY-ESO-1 and MAGEA expression, nor between NY-ESO-1 and presence of CD8+ lymphocytes; a weak relationship was found between MAGEA and CD8 (p value<0.05) (Additional file 1: Supplementary Table 1). ER, PR and HER-2 were also analysed but were not helpful in predicting recurrence (Table 4). Radiotherapy was not associated with development of invasive disease or with NY-ESO-1 expression, so was not considered a true confounder of the aforementioned association (Supplementary Table 2).

**Table 4:** Markers by ER/PGR/HER2 status

Marker characteristics (N=42)	ER positive			P-value*	PGR positive			P-value*	HER2 positive			P-value*
	No (n=11)	Yes (n=25)	Missing (n=6)		No (n=15)	Yes (n=21)	Missing (n=6)		No (n=25)	Yes (n=8)	Missing (n=9)	
**NY-ESO-1**												
Negative (n=25)	7	14	4	0.4658	10	11	4	0.3153	14	6	5	0.2151
Positive (n=16)	4	11	1		5	10	1		11	2	3	
Missing (n=1)	0	0	1		0	0	1		0	0	1	
**MAGEA**												
Negative (n=34)	7	21	6	0.1569	9	19	6	0.0261	22	3	9	0.0019
Positive (n=7)	3	4	0		5	2	0		3	4	0	
Missing (n=1)	1	0	0		1	0	0		0	1	0	
**CD8**												
1+ (n=18)	2	10	6	0.46	3	9	6	0.0858	11	1	6	0.0246
2+ (n=9)	3	6	0		3	6	0		8	1	0	
3+ (n=13)	6	7	0		9	4	0		5	6	2	
Negative (n=1)	0	1	0		0	1	0		1	0	0	
Missing (n=1)	0	1	0		0	1	0		0	0	1	
**CD8 recoded**												
Negative or 1+ (n=19)	2	11	6	0.06	3	10	6	0.03	12	1	6	0.03
2+ or 3+ (n=22)	9	13	0		12	10	0		13	7	2	
Missing (n=1)	0	1	0		0	1	0		0	0	1	

*P-values were computed using a permutation chi square test using 10,000 permutations with missing values excluded.

## DISCUSSION

At present it is difficult to predict which cases of DCIS will recur. There have been several studies suggesting that HER2, p16, COX-2 in combination with Ki67 and histologic appearance will give some prognostic information, but these tests are not sufficiently accurate to predict outcome of DCIS in individual cases [11, 12, 13].

Here, we show that expression of NY-ESO-1 predicts those patients who will not subsequently develop invasive breast cancer, at least over the 10 year follow up period. NY-ESO-1 was shown to elicit spontaneous antibody and T-cell responses in a proportion of cancer patients whose tumours express this antigen and it constitutes one of the most immunogenic CT antigens identified so far [14].

The significant association we found between the expression of NY-ESO-1 and the lack of progression of the DCIS lesions may be due to the ability of the immune system to protect against cancer development by detecting (but not necessarily destroying) abnormal cells. NY-ESO-1 is expressed in a minority of, mainly ER negative breast cancers; this observation, together with the recent reports that NY-ESO-1 expression confers a good prognosis in triple negative IDC [15] may suggest that most NY-ESO-1 expressing breast cancer cells are indeed detected and removed by the immune system at an early stage in the cancer evolutionary process. This idea is supported by the fact that in most breast cancer studies of NY-ESO-1 and TILs, a heavy TIL cell infiltrate usually is seen in NY-ESO-1 expressing cancers [16].

Those cells that remain and express NY-ESO-1 may be thought of as being of little risk to the patient by the immune system; thus, the good prognosis associated with NY-ESO-1 expression may be a reflection of its expression in cells that do not also effect a ‘danger’ signal. This would explain the lack of association with CD8 positive cells, unlike the situation in IDC, where CT antigen expression often correlates with CD8 TIL infiltration [15, 16].

The function of NY-ESO-1 in cellular biology remains to be established, but there are data that suggest that MAGE may be an oncogene [17]. Interestingly in the present work expression of MAGEA shows a correlation with worse prognosis (Figure 1). MAGEA has also been studied extensively in IDC; the consensus is that it does not correlate with either survival or other breast cancer markers such as ER, PR, or HER2 [15]. Here we found that there was an association between MAGEA and HER2; the numbers are relatively small, however, and these markers should be examined in a much larger series of cases.

We analysed tumour grade, HER2, as well as ER in this series, but in view of the relatively small number of cases, we are at this point unable to determine whether the addition of these markers will add anything to the development of a prognostic score. A further, larger study will be needed to address this point.

Current breast cancer screening programmes have resulted in a great increase in the number of DCIS cases diagnosed: thus, many patients are undergoing breast surgery that, in some cases may not be warranted. Future studies will show whether CT antigen expression, perhaps in combination with other markers, can help predict which patients will ultimately develop breast cancer, and who therefore need potentially disfiguring surgery.

## MATERIALS AND METHODS

### Patients and samples

We systematically examined the cases of DCIS diagnosed at one institution (Charing Cross Hospital, London) with a mean follow up time of 10.54 (SD: 5.29) years**.** All cases of surgically resected DCIS during 1992-2006 were reviewed in this study and followed-up for subsequent IDC development. Age matched controls with similar follow-up time, consisting of patients with DCIS who remained disease-free, were also selected. Clinical samples were de-identified and obtained without individual consent under a protocol approved by the Charing Cross Hospital Institutional Review Board. All patients were treated with wide local excision and 36/42 (86%) patients received radiotherapy (Table 1) following surgery.

### Histology and immunohistochemistry

Archival H and E slides of all cases were reviewed to ensure the diagnosis of DCIS. Several new 5 micron sections were then cut from paraffin embedded blocks. One was stained with H and E to ensure the continued presence of the target lesion.

NY-ESO-1 (clone E978) and MAGEA (clone 6C1) were detected by IHC using previously validated and described reagents and methods [9, 10] Infiltrating CD8+ cells were demonstrated by IHC using C8/144B antibody as previously described [8]. Repeat immunostaining for ER, PR and HER2 was carried out as described previously [8].

### Statistical analysis

Descriptive statistics were conducted for all demographic and other study variables and included mean and ± standard deviation (SD) for continuous variables and count and percentages for categorical ones. Univariate statistics were performed comparing marker variables to Invasive Breast Cancer, to ER/PgR and HER2 status and to each other using a permutation chi square test with 10,000 permutations. Furthermore, univariate logistic regression models were first used to estimate the effect of each marker on the risk of development of Invasive Breast Cancer using recurrence as a categorical variable and then univariate cox proportional hazards regression were used incorporating time to recurrence to estimate probability of recurrence by marker status.

Descriptive Kaplan Meier curves were also used with the Log-rank test (LRT) comparing the survival distributions, with results shown in the figure heading and in (Supplementary Table 1). Potential confounding by DCIS type, age and radiotherapy was also assessed for each of the markers using a permutation chi square test or a permutation Kruskal Wallis test with 10,000 permutations as dictated by the categorical or continuous nature of the variables.

## CONCLUSIONS

In summary, we have found a ‘protective effect’ of NY-ESO-1 expression in DCIS cases that appears to confer, as in primary breast cancer, a good prognosis. This result needs to be confirmed in a much larger series of patients before NY-ESO-1 immunostaining could be used to stratify patients to assist clinical management.

Additionally, as a result of its high immunogenicity, NY-ESO-1 based vaccines have been tested in multiple trials that have demonstrated their ability of eliciting T-cell and antibody responses [4, 14]. Anti-cancer vaccines based on NY-ESO-1 may be beneficial in the management of DCIS patients to boost pre-existing immunity to NY-ESO-1, which may support T cell expansion and activation and help overcome immunosuppressive elements within the tumor, resulting in improved tumour rejection.

## SUPPLEMENTARY TABLES



## Abbreviations

DCIS: ductal-carcinoma-in-situ
CT: Cancer Testis
ER: Estrogen Receptor
PR: Progesterone Receptor
LRT: Log-rank test
IHC: Immunohistochemistry
IDC: invasive ductal carcinomas
