# Supplementary material for: NY-ESO-1 expression in DCIS: A new predictor of good prognosis
Source: Oncoscience. 2017 Apr 28;4(3-4):33–40. doi: 10.18632/oncoscience.348 (PMC5441475; doi:10.18632/oncoscience.348)
Supplement: Supplementary file 1 [file oncoscience-04-0033-s001.pdf]

NY-ESO-1 expression in DCIS: A new predictor of good prognosis

SUPPLEMENTARY TABLES

Supplementary Table 1: Univariate association between the markers

Marker characteristics (N=42)		NY-ESO-1_E978_IHC			MAGEA_6C1_IHC		
		Negative (n=25)	Positive (n=16)	Missing (n=1)	Negative (n=34)	Positive (n=7)	Missing (n=1)
**NY-ESO-1_E978_IHC**	Negative (n=25)				20	4	1
	Positive (n=16)				13	3	0
	Missing (n=1)				1	0	0
**P-value***						0.6686	
**MAGEA_6C1_IHC**	Negative (n=34)						
	Positive (n=7)						
	Missing (n=1)						
**P-value***							
**CD8_IHC**	1+ (n=18)	12	5	1	17	0	1
	2+ (n=9)	5	4	0	7	2	0
	3+ (n=13)	7	6	0	8	5	0
	Negative (n=1)	1	0	0	1	0	0
	Missing (n=1)	0	1	0	1	0	0
**P-value***			0.7414			0.0415	
**CD8_IHC recoded**	Negative or 1+ (n=19)	13	5	1	18	0	1
	2+ or 3+ (n=22)	12	10	0	15	7	0
	Missing (n=1)	0	1	0	1	0	0
**P-value***			0.1892			0.0016	

*P-values were computed using a permutation chi square test using 10,000 permutations with missing values excluded.

Supplementary Table 2: Checking for confounding by age, radiotherapy or DCIS type

Marker characteristics	DCIS type N		P-value*	Age Mean (SD)	P-value**	Radiotherapy		P-value*
	Low or Intermediate (n=27)	High (n=12)				No (n=6)	Yes (n=36)	
**NY-ESO-1_ E978_IHC ^**								
Negative (n=25)	14	8	0.1475	72.76	0.1493	4	21	0.6631
Positive (n=16)	13	3		75.67		2	14	
**MAGEA_6C1_ IHC ^**								
Negative (n=34)	23	8	0.4231	74.93	0.4224	4	30	0.3533
Positive (n=7)	4	3		68.81		2	5	
**CD8_IHC ^**								
1+ (n=18)	10	5	0.2978	75.99	0.737	2	16	0.6039
2+ (n=9)	8	1		75.3		1	8	
3+ (n=13)	7	6		70.18		3	10	
Negative (n=1)	1	0		80.6		0	1	
**CD8_IHC recoded^**								
Negative or 1+ (n=19)	11	5	0.7312	76.2	0.6813	2	17	0.3897
2+ or 3+ (n=22)	15	7		72.3		4	18	

*P-values were computed using a permutation chi square test using 10,000 permutations with missing values excluded.
^Note missing values were excluded from the table.
**P-values were computed using a permutation Kruskal Wallis test using 10,000 permutations with missing values excluded.
